# Human Coronavirus OC43 as a Low-Risk Model to Study COVID-19

**DOI:** 10.3390/v15020578

**Published:** 2023-02-20

**Authors:** Mi Il Kim, Choongho Lee

**Affiliations:** College of Pharmacy, Dongguk University-Seoul, Goyang 10326, Republic of Korea

**Keywords:** COVID-19, human coronavirus, biosafety level, OC43, low-risk model

## Abstract

The coronavirus disease 2019 (COVID-19) pandemic has had irreversible and devastating impacts on every aspect of human life. To better prepare for the next similar pandemic, a clear understanding of coronavirus biology is a prerequisite. Nevertheless, the high-risk nature of the causative agent of COVID-19, severe acute respiratory syndrome coronavirus 2 (SARS-CoV-2), requires the use of a cumbersome biosafety level-3 (BSL-3) confinement facility. To facilitate the development of preventive and therapeutic measures against SARS-CoV-2, one of the endemic strains of low-risk coronaviruses has gained attention as a useful research alternative: human coronavirus OC43 (HCoV-OC43). In this review, its history, classification, and clinical manifestations are first summarized. The characteristics of its viral genomes, genes, and evolution process are then further explained. In addition, the host factors necessary to support the life cycle of HCoV-OC43 and the innate, as well as adaptive, immunological responses to HCoV-OC43 infection are discussed. Finally, the development of in vitro and in vivo systems to study HCoV-OC43 and its application to the discovery of potential antivirals for COVID-19 by using HCoV-OC43 models are also presented. This review should serve as a concise guide for those who wish to use HCoV-OC43 to study coronaviruses in a low-risk research setting.

## 1. Introduction

Despite the successful development and active administration of several commercially available vaccines and antivirals, the suffering inflicted by the coronavirus disease 2019 (COVID-19) pandemic seems likely to continue for the foreseeable future. The emergence of more contagious variants of severe acute respiratory syndrome coronavirus 2 (SARS-CoV-2) is further aggravating the already-devasted healthcare systems around the world. The classification of SARS-CoV-2 as a biosafety level 3 (BSL-3) pathogen due to its relatively high fatality rate has restricted its accessibility to the scientific community for research purposes. This has been one of the biggest hurdles to overcome in the prompt development of effective vaccines and therapeutics against COVID-19. In this regard, human coronavirus OC43 (HCoV-OC43) has gained new attention as an attractive and safe model to study coronaviruses in the low-risk research setting (BSL-2). To better understand its utility as an alternative to SARS-CoV-2 research, the history and classification of HCoV-OC43 together with the clinical manifestations associated with its infection are first summarized. The characteristics of its viral genomes and genes are also presented in comparison with SARS-CoV-2. Then, its evolution process in the context of interspecies and intraspecies perspectives is further explained. The host–virus interaction sections cover the host factors necessary to support the viral life cycle of HCoV-OC43, followed by a description of the innate—as well as adaptive—immunological responses to HCoV-OC43 infection. The development and application of in vitro and in vivo models to study HCoV-OC43 and the discovery of potential antivirals for COVID-19 by using HCoV-OC43 models are also discussed.

## 2. Classification of Human Coronaviruses

In general, all coronaviruses are members of the *Coronaviridae* family of the order *Nidovirales*. Mammals including bats, cows, mice, birds, and humans serve as their natural hosts [1]. Based on sequence homology, they are further classified into four genera including alpha, beta, gamma, and delta coronaviruses. Endemic strains of human coronavirus such as 229E and NL63 are typical examples of alpha coronaviruses. The genus beta coronavirus is further divided into 2a (OC43 and HKU1), 2b (SARS), and 2c (middle east respiratory syndrome, MERS) coronaviruses [2,3,4]. Animal viruses such as mouse hepatitis virus (MHV) and bovine coronavirus (BCoV) also belong to the genus beta coronavirus. HCoV-229E, HCoV-NL63, HCoV-HKU1, and HCoV-OC43 are four typical co-circulating low-risk human coronaviruses. They are predominantly associated with mild infection of the upper respiratory tract [5]. They have become endemic respiratory coronaviruses since their introduction into the human population [2]. Due to their zoonotic origin, all these endemic strains are thought to have broken the interspecies barrier, resulting in altered tropism for humans [6]. With regard to the zoonosis of SARS-CoV-2, the origin of SARS-CoV-2 infection in the wild was proposed to begin from bats based on sequencing results of bat coronaviruses [7,8]. In the wild, reptiles, horses, camels, sheep, llamas, marine mammals, and badgers were suspected to be infected with SARS-CoV-2 with no experimental evidence [9]. Although some cases of SARS-CoV-2 infection in pigs, birds, and cows were reported, they all seem to be inconclusive or lack experimental evidence [9]. Interestingly, the transmission of SARS-CoV-2 from human to animals including cats, dogs, tigers, lions, and mink has also been confirmed [9]. As experimental models to study SARS-CoV-2, mice, hamsters, and ferrets are generally used for this purpose [9,10].

## 3. Brief History of HCoV-OC43

As explained previously, HCoV-OC43 is one of the seasonally circulating community-acquired coronaviruses, together with other three endemic strains. HCoV-OC43 was first discovered by using a human embryonic tracheal organ culture (OC) technique [11,12]. An examination of nasopharyngeal washings from an acute respiratory tract disease patient led to the recovery of multiple strains of infectious agents. Their sensitivity to ether indicated the requirement of a lipid-containing protein coat for productive infection [11,12]. Among them, two of the six strains, designated OC38 and OC43, were able to cause encephalitis when inoculated into newborn mice, highlighting their distinct neurotrophic pathogenesis despite their respiratory origin [13]. Due to the morphological resemblance to avian infectious bronchitis virus (IBV), HCoV-OC43 has initially been named an “IBV-like” virus [11,14]. A hemagglutination inhibition test was frequently utilized as a preferred method for its characterization [15]. In addition, owing to its close structural similarity to MHV, an adaptation of OC43 to the suckling-mouse brain was empirically tested and successfully utilized for its efficient propagation [12,13].

## 4. Clinical Manifestations of HCoV-OC43 Infection

In general, infection with human coronaviruses is responsible for common colds. Some patients show symptoms of diarrhea and enterocolitis. Roughly, 15 to 35% of all common colds are estimated to be attributed to coronavirus infection [12,16,17]. On occasion, they have been associated with neurologic disorders such as multiple sclerosis [18]. According to surveillance data, HCoV-OC43 has been regarded as the most frequently found strain responsible for coronavirus-associated mild colds [19]. In particular, among all respiratory viruses identified in respiratory infection samples, 4.7% turned out to be related to HCoV-OC43 [20]. Along with HCoV-229E and HCoV-NL63, HCoV-OC43 has also been shown to be a primary etiological agent for mild upper respiratory tract infections in children [12,20,21,22]. In line with this, a higher rate of infections with HCoV-OC43 has been consistently reported [23,24,25,26,27]. Although HCoV-OC43 infections are generally mild, subclinical, and self-limited [28], more severe respiratory tract infections including bronchiolitis and pneumonia have been reported in high-risk groups. These are infants, elderly individuals, and immunocompromised patients [29,30,31]. Despite the potential involvement of HCoV-OC43 in neurologic diseases, its association with neurotrophic etiology has never been validated [32,33]. The main symptoms of HCoV-OC43 infection are similar to respiratory tract infection symptoms like fever and cough [20]. Infection with other respiratory viruses has also been suggested as one of the contributing factors to the development of lower respiratory tract infections by HCoV-OC43 [20].

Unlike common community-acquired endemic human coronaviruses, recently emerged novel coronaviruses such as SARS-CoV-1, SARS-CoV-2, and MERS-CoV can cause a highly severe clinical course of infection and fatality mainly in elderly patients, particularly those with previous chronic medical conditions [34]. However, given the gradually decreased clinical severity shown in recently reported variants, SARS-CoV-2 seems to be in the process of establishing itself as one of these endemic human respiratory coronaviruses [2]. In addition, several reports suggest a protective role of pre-existing cross-immunity against endemic human coronaviruses in SARS-CoV-2 patients [35]. Therefore, understanding the mutual relationship between high- and low-risk coronaviruses will be critical not only for the development of effective countermeasures against current pandemics but also for the correct prediction of the evolutionary direction of current high-risk coronaviruses in the future.

## 5. Genome Structure of HCoV-OC43

**Figure 1:**
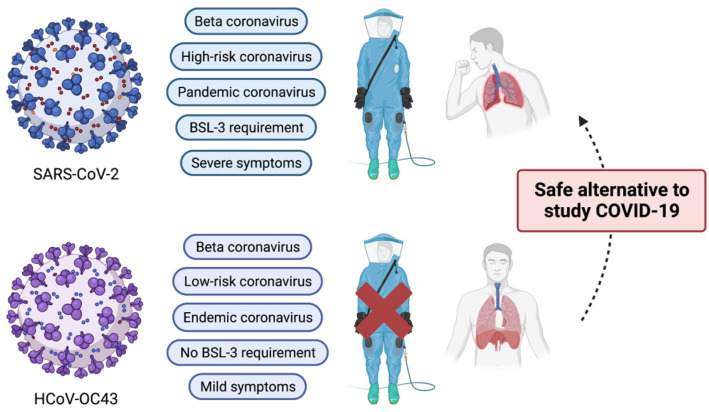
Comparison of major characteristics of SARS-CoV-2 and HCoV-OC43. Despite belonging to the same beta coronavirus, HCoV-OC43 is classified as an endemic coronavirus. In addition, due to its mild clinical symptoms, its biology can be studied under the BSL-2 research setting. Therefore, HCoV-OC43 can serve as a good research alternative to SARS-CoV-2. This illustration was created with BioRender.com.

**Figure 2:**
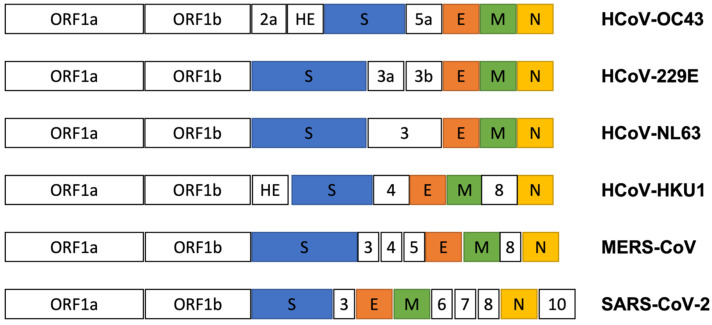
Comparison of abbreviated genomic maps of HCoV-OC43 with other coronaviruses including HCoV-229E, HCoV-NL63, HCoV-HKU, MERS-CoV, and SARS-CoV-2 (not to the scale). Used acronyms are as follows. ORF; open reading frame, HE; hemagglutinin-esterase, S; spike, E; envelope, M; membrane, N; nucleocapsid. Number-only genes (2a, 5a, 3a, 3b, 3, 4, 5, 6, 7, 8, 10) indicate nonstructural genes. Some in-between nonstructural genes from SARS-CoV-2 were not shown for purposes of better comparison.

Structurally, the single-stranded RNA genome of coronavirus is encapsidated in a helical nucleocapsid [28]. In general, the initial portion of the coronavirus genome encodes two long open reading frames (ORF1a and 1b), which generate sixteen non-structural (NS) proteins necessary for viral replication. Four structural proteins and accessory proteins (NS2a and NS5a) are translated from their separate subgenomic RNAs. Viral structural proteins include spike (S), envelope (E), matrix (M), and nucleocapsid (N) proteins [36] (Figure 1). The complete genome sequence of SARS-CoV shares 53.1% identity with that of HCoV-OC43, suggesting its significant relation to HCoV-OC43 [31,37]. In particular, comparisons with the SARS-CoV nucleotide and amino acid sequences suggest that HCoV-OC43 shares highly homologous motifs necessary for viral replication and pathogenesis [31]. In this regard, HCoV-OC43 can be regarded as a good alternative model to study SARS-CoV biology without the need for level three containment facilities [31]. However, unlike SARS-CoV, HCoV-OC43 and HCoV-HKU1 contain extra NS2a and hemagglutinin-esterase (HE) genes in front of the S gene (Figure 2). The relatively high homology (92%) of the HCoV-OC43 NS2a protein with that of BCoV strongly suggests its bovine origin [38]. As shown in Figure 2, the 3′ terminal region of the HCoV-OC43 genome encodes the NS2a, HE, S, NS5a, E, M, and N genes in order [28,39,40,41]. The exact roles of the accessory proteins such as NS2a and 5a in the life cycle of HCoV-OC43 are unknown, although they are suggested to be associated with the host immune evasion process [42,43].

Thus far, only three papers have described the functions of the NS proteins of HCoV-OC43 in the context of HCoV-OC43 biology [38,44,45]. Mounir et al. characterized the nucleotide and amino acid sequences of NS2 [45]. Labonte et al. confirmed the expression of the NS2 protein in HCoV-OC43-infected HRT-18 cells. Dolliver et al. showed the inhibitory ability of the NS1 protein of HCoV-OC43 against stress granule formation [44]. Since most of early studies examined the functions of the structural proteins of HCoV-OC43, we focus on the description of characteristics of the structural proteins of HCoV-OC43 in the following section.

## 6. Characteristics of Structural Proteins of HCoV-OC43

### 6.1. Hemagglutinin-Esterase

Only group 2a coronaviruses have an envelope-associated HE gene [46]. Among the structural proteins expressed by HCoV-OC43, HE possesses several unique characteristics, which are absent in SARS-CoVs [12]. The HE gene of HCoV-OC43 is hypothesized to have borrowed from the influenza C virus by heterologous recombination through a common ancestral origin [47,48]. Morphologically, HE is observed to be interspersed as stubby homodimeric projections in the HCoV-OC43 virion [49,50,51]. In general, HE possesses dual receptor-binding and receptor-degrading functions (Table 1). A lectin domain specific for O-acetylated sialic acid and a sialate-O-acetylesterase domain are required to exert these two functions in HE [52,53,54,55,56]. In HCoV-OC43, the inhibition of the acetyl esterase activity of HE led to the blockage of the production of infectious viruses and subsequent virus spread in cell culture [57]. Whereas the carbohydrate-binding activity of HE is strictly maintained in BCoV [58], the progressive accumulation of mutations has rendered HCoV-OC43 incapable of this function. This seems to be a strategy of HCoV-OC43 for adaptation to a new host, the human [59]. Despite this loss, HCoV-OC43 was still able to execute a reversible virion–sialic acid attachment strategy similar to the influenza virus by using its S protein [60]. In this regard, the S and HE proteins of HCoV-OC43 appear to have coevolved into functionally interdependent viral structural proteins to maximize their host attachment and release abilities [60]. 

### 6.2. Spike Protein

The amino acid sequence of the S protein of HCoV-OC43 demonstrates 91% similarity with that of BCoV [63]. Only the S protein was able to agglutinate chicken erythrocytes; thus, the S protein can be regarded as the main determinant for hemagglutination by HCoV-OC43 (Table 1) [61]. In regards to carbohydrate-binding specificity, the S protein of HCoV-OC43 has shown a high binding affinity to carbohydrates with alpha 2,6 linkage [61]. For most coronaviruses, the C-terminal domain of the S protein functions as the protein receptor-binding domain (RBD), whereas the N-terminal domain of the S protein was shown to mediate sugar receptor-binding in HCoV-OC43 [64]. 

### 6.3. Envelope Protein

The homo-oligomerization of coronavirus structural E protein leads to the formation of ion channels (Table 1) [62]. Functionally, the HCoV-OC43 E protein is required not only for the production of infectious viruses but also for the development of neurovirulence in animals [62]. In addition, the HCoV-OC43 E protein and its transmembrane domain were shown to be essential for efficient replication in the murine central nervous system (CNS) [62]. Of note, the NS5a protein of HCoV-OC43 was also shown to be another virally encoded ion channel. It was demonstrated to be involved in virion morphogenesis and pathogenesis [65].

### 6.4. Membrane Protein

The amino acid sequence of the M protein of HCoV-OC43 shows 94% homology with that of BCoV. Its N-terminal domain possesses six potential O-glycosylation sites [45]. The nature of this O-glycosylation was further verified by the failure to inhibit the glycosylation of the M protein by treatment of tunicamycin [45]. In line with its structural functionality, the M protein has also been shown to be required for virion morphogenesis (Table 1) [45].

### 6.5. Nucleocapsid

The nucleotide and amino acid sequences of the N gene of HCoV-OC43 displayed high homology to those of BCoV [66]. The binding of the N protein to the viral RNA genome is necessary for the formation of a helical nucleocapsid that is coated by the viral envelope (Table 1) [1]. An internal N protein is thought to be in touch with the internal portion of the M protein [67]. The N-terminal of the HCoV-OC43 N protein has three RNA-binding regions (residues 1–173). The C-terminal region (residues 301–448) of the HCoV-OC43 N protein does not have RNA-binding activity. Most of this region is responsible for the oligomerization of the HCoV-OC43 N protein [68]. The N-terminal domain of the N protein from HCoV-OC43 is enriched with positively charged amino acids. This domain has been found to mediate RNA-binding to form the RNA genome¬–capsid complex [69].

## 7. Evolution of HCoV-OC43

The mutating abilities of coronaviruses and their high recombination frequencies have been the main driving forces for introducing a new coronavirus from animals to humans [70,71]. In particular, cattle and swine have served as intermediate hosts for interspecies transmission [28,72]. Therefore, its constant genetic recombination with different coronaviruses in animal populations is thought to have contributed to its evolution into the current form of HCoV-OC43 [36,73].

Understanding the adaptive process of an animal coronavirus to a human host is a prerequisite to revealing the origin of the COVID-19 pandemic [28]. Remarkable antigenic and genetic similarities have been consistently found between HCoV-OC43 and BCoV [28,74,75,76,77]. In essence, HCoV-OC43 appears to have emerged from a spillover of BCoV [60]. The sequence analysis of the S gene of BCoV and HCoV-OC43 indicates a high probability of a recent host jump from animals to humans [28]. Specifically, HCoV-OC43 was postulated to have diverged from BCoV in 1890 [28]. The absence of 290 nucleotides from the S gene of HCoV-OC43 compared with that of BCoV seems to have been necessary for HCoV-OC43 to adapt to a new host. However, despite their high levels of similarity, HCoV-OC43 has maintained several unique features distinct from BCoV. First, two coding regions located downstream of the S gene of BCoV are missing in HCoV-OC43 [78]. In addition, HCoV-OC43 has also abandoned the HE lectin function as an adaptive effort to become a human pathogen [60]. This carbohydrate-binding activity seems to be provided by the S protein, instead. In addition to interspecies evolution, the co-circulation of numerous recombinant variants seems to give rise to the intra-species variability in HCoV-OC43 [79]. In particular, the genetic instability of the S gene seems to be pivotal for genotype persistence in human populations [64]. The generation of a novel genotype by natural recombination appears likely to continue for the foreseeable future [41]. In addition, regions of the viral S protein of HCoV-OC43 are continually exposed to human humoral immunity. Since HCoV-OC43 will undergo adaptive evolution in these regions, these adaptive alterations in antigenic regions of the virus will require the continual reformulation of already-made vaccines [80].

## 8. Host Interactions with HCoV-OC43

### 8.1. Virus Entry Factors

Sialic acids are found as terminal sugars of oligosaccharides present on many glycoproteins and glycolipids on cell surfaces [81]. Like influenza viruses, HCoV-OC43 utilizes a sialic acid as an entry receptor (Table 2) [6,82,83,84]. Since this receptor binding is mediated by the S protein, it is regarded as the main determinant for coronavirus host specificity [6]. Although two studies have reported the major histocompatibility complex (MHC) class I as another potential host receptor for HCoV-OC43 (Table 2) [18,85], there were no follow-up studies to validate this discovery. As explained previously, HCoV-OC43 and BCoV exhibit remarkable antigenic and genetic similarities. In addition, they both can agglutinate erythrocytes by attachment to N-acetyl-9-O-acetylneuraminic acid [81]. The interaction between HCoV-OC43 and N-acetyl-9-O-acetylneuraminic acid is required not only for erythrocytes agglutination but also for target cell infection with a preference for the alpha 2,6 linkage [61,81]. After attachment, HCoV-OC43 has been shown to employ caveolin-1-dependent endocytosis for viral entry and dynamin-dependent budding for viral exit (Table 2) [86]. In particular, the internalization of a virus particle was shown to require actin cytoskeleton rearrangements [86]. 

As previously mentioned, the most striking difference between HCoV-OC43 and SARS-CoV-2 might be the usage of host receptors for their entry. HCoV-OC43 takes advantage of 9-O-acetyl sialic acid for its initial attachment to the host cell [6,82,83,84] and engages with MHC class I to reach the host cytoplasm [18,85], while SARS-CoVs mainly depends on angiotensin converting enzyme 2 (ACE2) for this same viral process [91]. Paradoxically, infection with HCoV-OC43 can even induce the expression of MHC class I to boost its entry [92]. Based on these observations, HCoV-OC43 does not seem to be the ideal alternative to study the entry process of SARS-CoVs. Therefore, extra caution needs to be taken to interpret the virus entry-related data obtained from the HCoV-OC43 system for their potential application to the development of vaccines and antivirals against SARS-CoVs.

### 8.2. Intracellular Host Factors

After viral entry, coronaviruses take advantage of various host factors to support their viral life cycle inside infected cells. To identify these necessary host factors, several protein¬–protein interaction studies were conducted. Davies et al. reported strong interaction of viral NS2 and NS4 from HCoV-OC43 and SARS-CoVs with proteins localized at mitochondria-associated ER membranes (Table 2) [87]. This study further confirms the importance of the ER as the major subcellular organelle to support the genome replication of coronaviruses. To validate a recently published SARS-CoV-2 protein interactome, Hoffman et al. designed a CRISPR¬–Cas9 library targeting 332 proteins [88]. According to this study, they found virus-specific differences in Rab GTPase requirements and glycosylphosphatidylinositol anchor biosynthesis. They also identified multiple pan-coronavirus factors involved in cholesterol homeostasis. They seem to be necessary for virus assembly and trafficking (Table 2) [88]. Another group of researchers also conducted genome-wide CRISPR screening by using HCoV-OC43, HCoV-229, and SARS-CoV-2 [89]. In this study, they confirmed the already-known viral entry factors ACE2 (SARS-CoV-2), aminopeptidase N (HCoV-229E), and glycosaminoglycans (HCoV-OC43). In addition, this study also identified endosome maturation, phosphatidylinositol phosphate biosynthesis, and cholesterol homeostasis as essential host pathways for all three coronaviruses. These host processes seem to be linked to virus assembly and trafficking processes [89]. In line with this, pharmacological inhibition of phosphatidylinositol kinases and cholesterol homeostasis led to the attenuated propagation of these coronaviruses (Table 2) [89]. Schneider et al. also conducted similar genome-scale CRISPR knockout screening by using HCoV-OC43, HCoV-NL63, HCoV-229, and SARS-CoV-2 [90]. According to this study, they identified the vacuole membrane protein 1 (VMP1), transmembrane protein 41B (TMEM41B), and TMEM64 as host factors required for infection by all these coronaviruses. These host factors were proposed to be involved with ER membrane remodeling (Table 2) [90]. All of these screening results further validate the utility of HCoV-OC43 as an alternative research tool to study high-risk SARS-CoVs. 

### 8.3. Host Immunological Responses

Innate and adaptive host immune responses to virus infection are critical components of the host immune system to maintain a virus-free host environment. Therefore, as a countermeasure to the host defense, a viral immune evasion strategy is also essential for virus survival inside the host cell. The secretion of interferon (IFN) by infected host cells after virus recognition plays a central role in establishing innate immune defense in neighboring cells against virus infection. In general, interferons are detrimental to a virus due to the activation of MHC antigen processing and the antiviral action of various interferon-stimulated genes (ISGs). Paradoxically, IFN-γ was shown to enhance HCoV-OC43 infection of neuronal cells by increasing the expression of MHC class I, which was proposed as another viral entry receptor [92]. This increased host cell susceptibility to HCoV-OC43 infection was further validated by a receptor blockade with a monoclonal antibody specific for MHC class I [92]. In this regard, HCoV-OC43 seems to be able to take advantage of the virus-induced antiviral actions of IFNs. On the other hand, the expression of the HCoV-OC43 N protein led to the potentiation of NF-kB activation [93]. Mechanistically, the binding of the N protein to microRNA 9 (miR-9) was shown to relieve the negative regulation of NF-kB by miR-9 [93]. In support of the positive role of IFNs in the coronavirus life cycle, all three types of IFNs including IFN-α, IFN-γ, and IFN-λ were shown to efficiently promote HCoV-OC43 infection [94]. This enhancement of HCoV-OC43 infection by IFNs seems to be related to the increased availability of human IFN-induced transmembrane protein 2 (IFITM2) and IFITM3, which were also shown to serve as additional entry factors for HCoV-OC43 [94]. In regards to the blockage of induction of innate immunity by HCoV-OC43 infection, the transcriptional activities of interferon-sensitive response elements (ISREs), as well as IFN-β and NF-κB promoters, were significantly inhibited by HCoV-OC43 accessory proteins such as NS2a and NS5a and structural proteins such as M and N [42,43]. In line with this negative modulation of innate immunity by HCoV-OC43, Loo et al. also reported negligible innate immune activation by HCoV-OC43 infection in differentiated primary human bronchial epithelial cells [95]. 

With regard to the adaptive immunity induced by HCoV-OC43, SARS-CoV-2 antibodies cross-reacted with the S proteins of other beta coronaviruses [96]. From the clinical perspective, COVID-19 patients showed more severe disease progression when they had significantly lower levels of antibodies against the HCoV-OC43 N protein [97]. These results indicate the protective effects of previous infections with seasonal low-risk coronaviruses against COVID-19 [97]. Low pathogenic HCoVs strains such as OC43 and NL63 were also shown to induce T cells cross-reactive to SARS-CoV-2 [98]. In addition, memory CD8+ T cells specific for endemic coronavirus were also suggested to affect immune responses against SARS-CoV-2 [99]. This finding was remarkable considering a lower percentage (5.4%) of the epitope sharing of four endemic coronaviruses including 229E, HKU1, NL63, and OC43 with SARS-CoV-2. When the serum of SARS-CoV patients was examined, N peptide sequences were shown to play a critical role in cross-reactivity between HCoV-OC43 and SARS-CoV [100]. In general, a more severe clinical manifestation was observed following SARS-CoV-2 infection in older COVID-19 patients. This observation could be ascribed to the absence of pre-experienced T-cell immunity induced by endemic coronaviruses in elderly subjects [98]. Specifically, the C-terminal epitopes in the S protein of SARS-CoV-2 display a higher homology to the S proteins of low-risk coronaviruses such as 229E and OC43 [101]. Previous encounters with endemic coronaviruses might give rise to cross-reactive T cells specific for S proteins. Consequently, they were thought to play a protective role against COVID-19 pathogenesis [101]. In support of this finding, Mateus et al. also confirmed the presence of preexisting memory CD4+ T cells that are cross-reactive to SARS-CoV-2 and HCoV-OC43 [102]. Grifoni et al. also detected SARS-CoV-2-specific CD4+ T cells in unexposed cohorts. These data indicate the overlapping nature of T-cell recognition between low-risk coronaviruses and SARS-CoV-2 [103]. 

## 9. Neuropathology by HCoV-OC43 Infection

### 9.1. In Vitro Model

Human coronaviruses can infect some CNS-derived cells including astrocytoma, neuroblastoma, and the immortalized fetal microglial cell lines [104,105,106]. However, the infection of these neuronal cells failed to produce infectious HCoV-OC43 virions [107,108]. In general, the infection of glial cells by coronaviruses can lead to inflammation and induction of apoptosis, which are two relevant events for CNS pathologies [109]. Nevertheless, a causative connection between HCoV-OC43 and neurologic diseases remains unproven [110,111]. Continuous propagation of HCoV-OC43 in human neural cell cultures resulted in mutations in the viral S glycoprotein [112]. In particular, highly enhanced cell death in murine and human neuronal cells was observed by infection of HCoV-OC43 with two-point mutations in the S glycoprotein [113]. These data suggest the importance of S protein as a key determinant for HCoV-OC43-induced neuropathology.

### 9.2. In Vivo Model

Intracerebral or extraneural infection of suckling CD1 mice with HCoV-OC43 resulted in a neurotropic lethality in infected mice. High titers of the HCoV-OC43 virus were recovered from their brains. However, when mice grew older than 20 days, they became completely insusceptible to HCoV-OC43 inoculation. Mechanistically, the immune response seems to be ascribable to the development of resistance to HCoV-OC43 infection, in part [114]. BALB/c mice were also shown to be susceptible to an acute and persistent HCoV-OC43 infection [115]. Acute encephalitis was observed by intracerebral injection of HCoV-OC43 into BALB/c mice, followed by neuronal cell death by necrosis and apoptosis [110]. Axonal transport appears to facilitate the neuron-to-neuron propagation of HCoV-OC43 in mice brains [116]. There seems to be a significant difference in the degree of neurovirulence between mouse-CNS-adapted versus tissue-culture-adapted HCoV-OC43, since the intranasal inoculation of 8-week mice with a mouse-CNS-adapted strain was uniformly fatal while the tissue-culture-adapted strain was not [117]. The surface glycoprotein is thought to play a major role in the development of virulence in coronavirus infections. As mentioned previously, the acquirement of mutations in the S glycoprotein during viral persistence was able to produce more severe neurovirulence phenotypes when inoculated into susceptible mice. For example, the mutant HCoV-OC43 with S glycoprotein point mutations (H183R and Y241H) was able to give rise to a heightened unfolded protein response and attenuated protein translation. Therefore, this mutant virus turned out to be more neurovirulent in mice [118]. In addition, this mutant HCoV-OC43 was able to induce a highly deteriorated neuropathology in BALB/c mice. This mutant HCoV-OC43 was also involved with increased viral spread and enhanced T-cell infiltration, as well as the enhanced production of proinflammatory cytokines [112]. Mechanistically, receptor-interacting protein kinase 1 (RIP1) and mixed lineage kinase domain-like (MLKL) genes seem to play a pivotal role in the neuronal cell death induced by HCoV-OC43 [113]. A reporter HCoV-OC43 strain expressing renilla luciferase induced fatality in suckling mice after intranasal inoculation [119]. Le Coupanec et al. suggested the strong association of efficient cleavage of the S protein with HCoV-OC43-induced neurovirulence [120].

### 9.3. Clinical Model

HCoV-OC43 has been detected in the brain of pediatric patients with acute disseminated encephalomyelitis or fatal encephalitis [121,122]. This HCoV-OC43-induced fatal encephalitis was also diagnosed in an infant with aplastic thymus and chronic T-cell lymphopenia [123]. However, an etiologic connection between HCoVs and neurotropic diseases is not yet clear [111]. Although a higher prevalence of HCoV-OC43 in multiple sclerosis patients than in controls was observed [124], no significant differences were found between the multiple sclerosis patients and the normal subjects in their antibody titer to HCoV-229E and HCoV-OC43 [125].

## 10. Discovery of Antiviral Candidates by Using HCoV-OC43

Several antiviral candidates for coronavirus infection were identified by using HCo-OC43 to treat endemic coronavirus infections. Since the COVID-19 pandemic, the utility of HCoV-OC43 has been further applied to the discovery of anti-SARS-CoV-2 compounds due to its low-risk potential. Degiaggi et al. found that phosphatidyl-serine was able to inhibit HCoV-OC43 (Table 3) [126]. Cystatin C and D, potent inhibitors of cysteine proteases such as papain and cathepsin B, were also shown to inhibit HCoV-OC43 at its physiologic concentration (Table 3) [127,128]. Chloroquine, which once showed promise as a new class of SARS-CoV-2 drugs, potently suppressed HCoV-OC43 replication in vitro, with an IC_50_ of 0.33 μM by using the HCoV-OC43 reporter virus with a renilla luciferase gene (Table 3) [129]. Chloroquine was also able to reduce the mortality of HCoV-OC43-infected newborn C57BL/6 mice after transplacental or maternal milk delivery [130]. Emodin, an active component of several plants used in traditional Chinese medicine was able to inhibit the 3a ion channel of coronavirus SARS-CoV and HCoV-OC43 (Table 3) [131]. Meanwhile, memantine, an NMDA receptor antagonist was demonstrated to attenuate mortality rates and body weight loss in the HCoV-OC43-infected mice (Table 3) [132]. HTCC, a cationically modified chitosan, inhibited the replication of several endemic strains of human coronaviruses, including HCoV-NL63, HCoV-OC43, and HCoV-HKU1 replication in human airway epithelial cells, by disrupting the virus–receptor interactions (Table 3) [133]. The mechanism of the antiviral actions of this polymer seems to involve the blockage of virus entry into the host cell by interaction with the S protein [134]. Lycorine, a toxic crystalline alkaloid found in various *Amaryllidaceae*, was able to reduce the fatality of BALB/c mice by HCoV-OC43 by attenuating the viral spread in the CNS of infected mice (Table 3) [135]. Kim et al. reported that bis-benzylisoquinoline alkaloids were able to significantly inhibit HCoV-OC43-induced cell death at the early step of the virus life cycle (Table 3) [136]. Amiloride, which is a diuretic to treat hypertension, was shown to inhibit HCoV-OC43 replication via specific interactions with stem-loop structures of viral RNAs (Table 3) [137]. Tylophorine-based compounds and natural cardiotonic steroids such as cardenolides and bufadienolides were also able to inhibit HCoV-OC43 with nanomolar EC_50_ values (Table 3) [138]. Kurarinone, a flavanone from *Sophora flavescens* roots, inhibited HCoV-OC43 infection in human lung fibroblasts with an IC_50_ of 3.458 μM by impairing the virus-induced autophagy (Table 3) [139]. An anti-protozoal drug, emetine, has also been reported to have anti-coronavirus activity (Table 3) [140]. This compound was again found as an HCoV-OC43 inhibitor in HCT-8 cells (Table 3) [141]. The in vitro inhibitory capacity of the redox-active oxysterol 27-hydroxycholesterol against SARS-CoV-2 was further verified by using HCoV-OC43 (Table 3) [142]. Valinomycin, a naturally occurring dodecadepsipeptide, has been used as an antibiotic; its broad-spectrum antiviral activity was further confirmed by using HCoV-OC43 (Table 3) [40]. AT-527, a prodrug of a guanosine nucleotide analog was identified as a potent inhibitor of both SARS-CoV-2 and HCoV-OC43 in vitro (Table 3) [143]. Epigallocatechin gallate (EGCG), which is a green tea polyphenol, was able to decrease the 3CL-protease activity of HCoV-OC43 and HCoV-229E. In addition, EGCG treatment attenuated HCoV-OC43-associated cytotoxicity (Table 3) [144]. Lactoferrin is a multifunctional protein of the transferrin family. The broad-spectrum antiviral activity of lactoferrin was also confirmed by using HCoV-OC43. The binding of lactoferrin to heparan sulfate proteoglycans seems to be responsible for blocking viral attachment to the host cell (Table 3) [145].

## 11. Conclusions

In this review paper, we overviewed the history, classification, and clinical manifestations of HCoV-OC43. The characteristics of its genomes and genes and its evolution process were further explored. The essential host factors for the viral life cycle and the innate, as well as adaptive, immunological responses were also summarized. Finally, the available in vitro and in vivo systems to study HCoV-OC43 and its application to the discovery of potential antivirals for COVID-19 were also presented. HCoV-OC43 should provide a valuable tool, not only for the general study of coronavirus biology but also for the rapid identification of promising drugs against coronaviruses without the drawbacks of biosafety level 3 confinement.

## Figures and Tables

**Table 1:** Major characteristics of structural proteins of HCoV-OC43.

Structural Protein	Key Function	References
Hemagglutinin-esterase	Receptor-binding and degradation	[52,53,54,55,56]
Spike protein	Receptor-binding and hemagglutination	[61]
Envelope protein	Ion channel formation	[62]
Membrane protein	Virion morphogenesis	[45]
Nucleocapsid	Helical nucleocapsid formation	[1]

**Table 2:** Host factors or processes identified necessary for the HCoV-OC43 life cycle.

Host Factor or Process	Steps of Virus Life Cycle	References
9-O-acetyl sialic acid	Host receptor binding	[6,82,83,84]
MHC class I	Host receptor binding	[18,85]
Caveolin-1-dependent endocytosis	Virus entry	[86]
ER structure	Virus RNA replication	[87]
Rab GTPase and glycosylphosphatidylinositol	Virus assembly and trafficking	[88]
Endosome maturation, phosphatidylinositol phosphate, and cholesterol homeostasis	Virus assembly and trafficking	[89]
VMP1, TMEM41B, and TMEM64	ER membrane remodeling	[90]
Dynamin-dependent budding	Virus exit	[86]

**Table 3:** Anti-coronavirus candidate molecules identified by using the HCoV-OC43 infection model.

Name	Experiment Type	EC_50_ (mM) *	References
Phosphatidyl-serine	in vitro	na	[126]
Cystatin C and D	in vitro	0.8	[127,128]
Chloroquine	in vitro and in vivo	0.33	[129]
Emodin	in vitro (inhibition of 3a ion channel)	<10	[131]
Memantine	in vivo	NA **	[132]
HTCC	in vitro	NA	[133]
Lycorine	in vivo	<5	[135]
Bis-benzylisoquinoline alkaloids	in vitro	<0.1	[136]
Amiloride	in vitro	>10	[137]
Tylophorine-based compounds	in vitro	0.1–1	[138]
Cardenolides and bufadienolides	in vitro	0.1–1	[138]
Kurarinone	in vitro	3.458	[139]
Emetine	in vitro	0.21	[140,141]
Oxysterol 27-hydroxycholesterol	in vitro	<10	[142]
Valinomycin	in vitro	6.15	[40]
AT-527	in vitro	2.2	[143]
EGCG	in vitro	14.6	[144]
Lactoferrin	in vitro	<50 mg/mL	[145]

* Effective concentration to reduce the amount of virus proliferation in half. ** Not available.

## Data Availability

Not applicable.
